# *Haematopinus suis* Infestation in Pig Farms in Busogo Sector, Rwanda

**DOI:** 10.1155/2023/9960745

**Published:** 2023-04-30

**Authors:** E. Niyonsenga, J. Twizerimana, M. F. Mwabonimana

**Affiliations:** ^1^Department of Animal Production, College of Agriculture, Animal Sciences and Veterinary Medicine (CAVM), University of Rwanda, Nyagatare Campus, P.O. Box 210, Nyagatare, Rwanda; ^2^School of Veterinary Medicine, College of Agriculture, Animal Sciences and Veterinary Medicine (CAVM), University of Rwanda, Nyagatare Campus, P.O. Box 210, Nyagatare, Rwanda

## Abstract

*Haematopinus suis*(*H. suis*) is a common ectoparasite of pigs and is economically important worldwide. *H. suis* is responsible for anemia and poor feed conversion rate that lead to poor growth in pig husbandry. This study assessed the prevalence and risk factors of *H. suis* in pigs through a cross-sectional survey in Busogo sector of Musanze district. Fifty-five (55) pigs, representing 10% of 555 pigs from 20 farms, were examined physically for the presence of *H. suis*, and a total number of 559 *H. suis* were collected from them in Busogo sector of Musanze district. Data were analyzed using the Statistical Package for the Social Sciences (SPSS). Results showed that out of 55 pigs, a total number of 35 pigs (63.6%), were found infested with *H. suis* in Busogo sector of Musanze district. The infestation by *H. suis* was associated with the farming system, animal breeds, animal's category, sex, pig hygiene, and piggery hygiene. Results showed a high (*P* < 0.05) prevalence in pigs reared in the intensive system (91.4%), whereas large whites were the most affected breed (60%). The prevalence of *H. suis* varied significantly (*P* < 0.05) among sexes, and females were the most affected (60%). Results related to pig hygiene revealed that all farmers were practicing washing skin three times per week, whereas only 60.0% of them were removing the bedding in their piggery. The study concluded that *H. suis* is present and remains a problem in the study area. Therefore, the study recommends to create farmer's awareness on the disease in pigs and its impact through training. Researchers should continue further studies on *H. suis* prevention with appropriate pig husbandry and management practices and the efficacy of acaricides used.

## 1. Introduction

The livestock sector plays an important role in sustainability of the world economy and attains food security in terms of protein availability and poverty alleviation [1, 2]. It can also provide livelihood and employment [3]. Most pigs are raised by smallholders in extensive, semiextensive, and intensive systems [4, 5]. Pig farming requires in general too much attention related to biosecurity measures, control, and management. Despite the biosecurity measures, ectoparasite such as Haematopinus suis remains a problem that is not well known and may be difficult to control. This parasite is one of the zoonotic ectoparasites of public importance and the common and largest prevalent ectoparasite of pigs in very cold environments and during high rainfall [6, 7]. *H. suis* is the only species of louse that infests pigs of all ages worldwide. It is a sucking louse which gets blood meals from the host through its penetrating mouth parts [8].

Most frequently, *H. suis* is responsible for persistent irritation, and biting witches may lead to reduction of feed efficiency with the possibility of anemia caused by loss of blood, hair loss, and disease spread [9, 10]. This happens mainly in pigs reared in poor infrastructures which lead to a low level of implementation of biosecurity measures [11]. In domesticated pigs, the parasites and their eggs can be found in the fence lines, dry feed, and mangers as well, with the ear, tail regions, and neck being the favored living site of the pig with restriction to the skin surface [12].

Studies conducted elsewhere around the word reported 66.7%, 2.5%, 14.5, 96.1%, 28.35%, and 32.4% of prevalence of infection by *H. suis* in Ghana, Germany, Botswana, Kenya, Nigeria, and Mozambique, respectively [12–17]. Another study by Meguini et al. [18] reported a prevalences of 25% and 28% in wild boars in Algeria, while in Tanzania, Braae *et al.* [6] reported 20% and 63% in pigs reared in the intensive system and the extensive system, respectively.

The following risk factors have been attributed to new purchased infected animals: lack of proper hygiene and disinfection, lack or inadequate parasite control, lack of housing, age of pigs, season, methods of cleaning [14, 19], and existence of infected pigs in the neighbouring farms [6].

Regardless of that, *H. suis* heavy infestations affect growth rates, cause anemia, and lead to economic loss in pig farming systems; there is no information of *H. suis* infestations in pigs in Rwanda. For that reason, this is the first work of *H. suis* infestation in Rwanda.

## 2. Materials and Methods

### 2.1. Study Sites and Questionnaire

**Figure 1:**
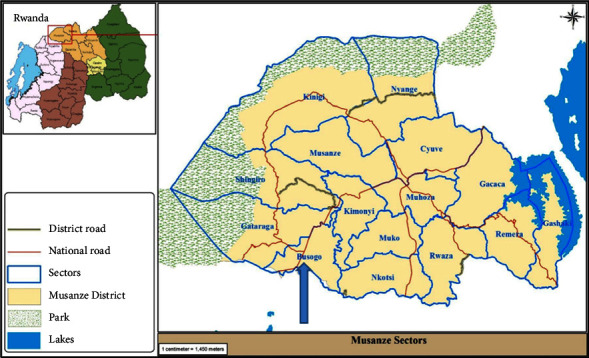
The map of Musanze district showing sector [21].

The study was conducted in Busogo Sector (Figure 1), located in Musanze District in Northern Province of Rwanda. Busogo Sector is made of 4 cells which are Gisesero, Sahara, Kavumu, and Nyagisozi. It has a mean altitude of 2300 m with the highest point being at 2800 m. The climate has a mean temperature of 16.7°C and much rain comprising between 1400 and 1800 mm. Busogo Sector has 4 seasons, which are divided as follows: a short dry season from mid-December to mid-February, a heavy rainy season from mid-February to the end of June, a heavy dry season extending from June to August, and a short rainy season from August to mid-December. Busogo Sector has mainly volcanic soil which is very permeable with low depth on mountains and moderate depth in lower altitude. This kind of soil is subjected to many erosion phenomena in the area of abrupt slope. The population is around 15,795 inhabitants, where 45.1% are male and 54.9% are female. The total surface area is 20.5 km^2^ with the population density of 787.8 inhabitants per km^2^. Most of the people in Busogo Sector are involved in agriculture, and the main crops grown are potatoes, maize, beans, and vegetables. The fauna is not dominating in those sectors because a good number of wild animals have migrated in the national parks because of hunters. On the side of flora, the natural vegetation is no longer present because of agricultural activities [20].

### 2.2. Study Design and Data Collection

A cross-sectional survey was conducted to collect qualitative data through face-to-face interviews using a structured questionnaire. The survey targeted pig's smallholder farmers who were identified and selected using the snowballing method at the cell level. However, the population size of pigs raised in Busogo sector was unknown; the larger sample size such as 10% of pigs was determined and used to determine the population size of a given pen. Data collected included the type of farming system, breeds, age, and hygienic management. At the animal level, observation and examination of the pig skin and skin lesions throughout the external surface of the body were performed and *H. suis* presence was also recorded.

### 2.3. Data Analysis

Data collected were recorded, encoded using Microsoft Excel 2007, and then exposed to the Statistical Package for Social Sciences (SPSS) version 21 for analysis. Descriptive statistics were used to summarize the social characteristics of farmers, farm characteristics, the infestation level, and the distribution of infested animals according to the farming system, breed, animal sex, and piggeries and pig hygiene. Results were interpreted using tables and figures.

## 3. Results

### 3.1. Social Characteristics of Farmers in Busogo Sector (*n* = 20)

A total of 20 pig smallholder farmers were interviewed in Busogo sector of Musanze District. Results from Table 1 shows that all (100%) farmers interviewed were within the age range between 60 and 70 years old and 85.0% of them were males. Out of the 20 smallholder farmers interviewed, 85.0% were involved in farming while the remaining 15% were involved in other activities as a primary occupation but took on farming as a secondary occupation. Majority 90.0% of them are practicing the intensive system, while 50.0% have experience of 1 to 2 years and another 50.0% have experience of 3 to 4 years. In the area studied, the educational level of the farmers shows that the majority 35.0% of farmers interviewed attended the university. Considering the location of majority of pig farmers interviewed, 55.0% are located in Sahara cells, while 40.0% and 5.0% are from Gisesero and Kavumu cells, respectively. The majority of farmers are using piggery constructed of wall timber (95.0%).

### 3.2. Farm Animal's Characteristics in Busogo Sector (*n* = 55)

**Figure 2:**
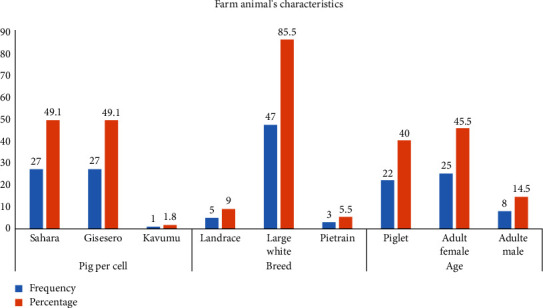
Animal characteristics in Busogo sector (*n* = 55).

Results (Figure 2) indicate that the total number of pigs by the cell was 27, 27, and 1, for Sahara, Gisesero, and Kavumu cells, respectively. Results also show that the majority of pigs reared in the study area was represented by the large white breed (85.5%) and adult female category (45.5%).

### 3.3. Farmers Infested by *H. suis* by Cells (*n* = 20)

Results (Table 2) indicate that 19 (54.3%), 15 (42.9%), and 1 (2.8%) pigs from 9, 6, and 1 farms were observed to be infested with Sahara, Gisesero, and Kavumu cells, respectively. This makes 35 pigs infested with three cells with Sahara being the cell with high number of pigs, 19 (54.2%) infested by *H. suis.*

### 3.4. Infestation by *H. suis* by Farming System Breeds and Sex in Different Cells (*n* = 35)

Results from Table 3 have shown that out of the 35 pigs infested by *H. suis*, pigs reared in the intensive system (91.4%) were the most infested, while based on the breed, the majority (48.6%) of them was large white breeds compared to Landrace and Pietrain breeds, and based on the animal sex, females (60.0%) were the most affected with *H. suis* than male pigs.

### 3.5. Farmer's Pig and Piggery Hygiene Practices

Results (Table 4) indicated that majority of farmers were washing their pigs three times per month (43.3%). With regard to piggery hygiene, 62.5%, 50.0%, 60.0%, and 75.0% of farmers are cleaning every day, using clean water, removing bedding, and using soaps and brushes, respectively.

## 4. Discussion

Out of 55 pigs, 35 (63.6%) of them were found infested with *H. suis* after practical examination of the skin. The prevalence of infestation was found more in pigs reared in the intensive system (90.0%) than the pigs maintained in individual houses in village condition (10.0%). According to the number of pigs observed, the results indicated that the Sahara cell has a high number of pigs infested by *H. suis* (54.4%) compared to Nyagisozi and Kavumu cells. The study elsewhere indicated that congestion and ineffective hygiene in piggeries are contributing factors in most farms to infestation by *H. suis.* The high number of infested pigs by *H. suis* in Sahara cells may be attributed to the fact that the Sahara cell has a high number of pigs reared in the intensive system which has been shown to have more pigs infested (91.4%) than other farming systems. This will require training farmers on the prevention and control of ectoparasites in pigs. These results differed from those reported by Kagira et al. [15] in Kenya and Islam et al. [5].

These results confirm the results by Islam et al., [22], and Islam et al., [7] reported high infestation by *H. suis* (100.0%) in intensive pigs in Bangladesh. These results do not agree with what Radostits et al. [23] reported, that pigs kept outdoors and those in poor body condition are more susceptible to *H. suis* than pigs in pens. Results suggest that there is a chance of spread of *H. suis* infestation in the housed farms which occurred by close contact between pigs or by contact with recently contaminated surfaces.

Results related to the sex of pigs indicated that the females are more attacked by *H. suis* than males. These results are in agreement with Kagira et al. [15], who reported the highest prevalence of *H. suis* in male (96.7) than in female (94.8) pigs in Kenya. The prevalence of *H. suis* was associated with the category of pigs, being highest in adult females but lowest in piglets and adult males. These corroborate with the results by Odo et al., [16] in Nigeria. The higher prevalence of *H. suis* in adult females could be attributed to the period the adult female pigs had been kept in the farm compared to the other categories of pigs. These results are in agreement with Samuel et al., [24] in USA, who reported that the higher prevalence of *H. suis* in sows could be related to the age of pigs.

Based on the piggery and pig's hygiene, the results revealed that piggery hygiene was done by cleaning with water (50.0%), removing bedding (60.0%), and using different materials such as soaps and brushes (75.0%), while at the animal level, pigs were washed three times per week. These results differed from those by Nsoso et al. [14], who reported that only 50% of farmers were removing the manure without any other form of disinfection or cleaning. This way of practice in pig husbandry may expose pigs to various parasites and the spread of many other pig diseases. These results suggest that the low hygiene in piggeries and on pigs could be the contributing factor of the high infestation by *H. suis.* Therefore, we would recommend a good and effective protocol of cleaning and disinfection of piggeries in Busogo sector, which will help in the disease control, reduction of antibiotic usage, risk of zoonosis, and reduction in disease.

## 5. Conclusion

The study concluded that *H. suis* is present in Busogo sector and affects more pigs reared in the intensive system with ineffective removal of bedding in piggery. Therefore, there are no data on the *H. suis* of domesticated pigs in Rwanda and yet the adoption of an intensive system is mandatory in pig husbandry in Rwanda. Studies to detect the *H. suis* parasites are needed because of their negative economic impact on the farmer's livelihood. However, appropriate steps should be taken by the government to create awareness on the parasite and its economic impact and promote the protocol of use of acaricides in the prevention of *H. suis* in pig husbandry. Further studies are recommended on the efficacy of acaricides used in the prevention of *H. suis.*

## Figures and Tables

**Table 1:** Farmer's social characteristics in Busogo sector (*n* = 20).

Variables	Statement	Frequency	Percentage
Age (years)	30–40	0	0
	41–50	0	0
	51–60	0	0
	61–70	20	100

Gender	Male	3	15
	Female	17	85

Location (cell)	Sahara	11	55
	Gisesero	8	40
	Kavumu	1	5

Education level	None	3	15
	Primary	4	20
	Secondary	6	30
	College/university	7	35

Occupation	Farming	17	85
	Others	3	15

Pig production experience (years)	1-2	10	50
	3-4	10	50
	5-6	0	0
	7-8	0	0

Farming system	Semiintensive	3	10
	Intensive	17	90
	Extensive	0	0

Material of the pen	Wall cemented	1	5
	Wall in timber	19	95
	Floor cemented	10	50
	Floor in timber	10	50

**Table 2:** The sample of a farm animal infested by *H. suis*.

Variable	Cells					
	Sahara		Gisesero		Kavumu	
	Farm	Animal	Farm	Animal	Farm	Animal
Number of farms and animals	1	2	1	4	1	1
	2	1	2	6		
	3	1	3	1		
	4	5	4	2		
	5	1	5	1		
	6	1	6	1		
	7	6				
	8	1				
	9	1				

Total *n*. (%)	9	19 (54.3%)	6	15 (42.9%)	1	1 (2.8%)

Total animals infested = 35						
Total farmers with animals infested = 16						

**Table 3:** Distribution of pigs infested by *H. suis* by the farming system, breed, and sex in different cells (*n* = 35).

Variables	Infestation results by cell			Total no. (%)
	Cells			
	Sahara	Gisesero	Kavumu	
Farming system				
Intensive	18	14	0	32 (91.4%)
Semiintensive	1	1	1	3 (8.6%)
Free range	0	0	0	0 (0%)
Breed				
Landrace	6	4	0	10 (28.6%)
Large white	8	8	1	17 (48.6%)
Pietrain	5	3	0	8 (22.8%)
Sex				
Male	8	6	0	14 (40%)
Female	11	9	1	21 (60%)

**Table 4:** Frequency of hygiene practice in farms in infected farms (*n* = 16).

Variables	Time/method	Response	Frequency	Percent (%)
Washing skin	Once	Yes	4	25.00
		No	12	75.00
	Twice	Yes	5	31.25
		No	11	68.75
	Three times	Yes	7	43.75
		No	9	56.25

Cleaning piggery	Cleaning with water	Yes	8	50.00
		No	8	50.00
	Remove of bedding	Yes	12	75.00
		No	4	25.00
	Cleaning with soap and brush	Yes	12	75.00
		No	4	25.00

Cleaning/week	Once	Yes	6	37.50
		No	10	62.50
	Every day	Yes	10	62.50
		No	6	62.50

## Data Availability

The data used are available in the manuscript.
